# Factors Affecting Growth and Survival of *Salmonella* in Onion Extracts and Onion Bulbs

**DOI:** 10.3390/foods14010001

**Published:** 2024-12-24

**Authors:** Emmanuel Kiplagat, Moazzameh Ramezani, Subas Malla, Luis Cisneros-Zevallos, Vijay Joshi, Alejandro Castillo

**Affiliations:** 1 Department of Food Science and Technology, Texas A&M AgriLife Research, College Station, TX 77843, USA; emmanuel.kiplagat@tamu.edu; 2 Texas A&M AgriLife Research & Extension Center at Uvalde, Uvalde, TX 78801, USA; mo.ramezani768@gmail.com (M.R.); subas.malla@ag.tamu.edu (S.M.); 3 Department of Horticulture, Texas A&M AgriLife Research, College Station, TX 77843, USA; lcisnero@tamu.edu

**Keywords:** *Salmonella*, food safety, onion, bulbs, extracts, inhibition, growth, survival, wounding, peeling

## Abstract

This study investigated the survival and growth of *Salmonella* in onion extracts and bulbs. The inhibition or retardation of *Salmonella* growth by extracts of red, white, and yellow onions was tested against the onion germplasm and exposure to different light spectra during curing. Separately, survival of *Salmonella* Newport was tested on red, white, and yellow onion bulbs on the external and internal onion layers with a syringe and needle. Onions exposed to blue, red, and white LED light during curing produced extracts with variable antimicrobial effects (*p* < 0.05), with those exposed to blue light showing the strongest inhibitory effect on red and white onions only. In survival studies, *Salmonella* inoculated on the outer scale was reduced by 1.2, >2.7, and >2.4 logs on red, white, and yellow onions, respectively, within 3 days, whereas it grew by 2.4, 2.6, and 2.8 logs inside red, white, and yellow onion bulbs, respectively, over 18 days. In separate trials, the outer layer again did not support the survival of *Salmonella* Newport. The a_w_ increased significantly from 0.51 to 0.58 in the outer scales and 0.96 to 0.98 for the fourth inner scales. Despite being rich in antimicrobial polyphenols, tissue integrity and water content may still promote *Salmonella* growth in onions.

## 1. Introduction

Bulb onions (*Allium cepa*) consist of red, white, and yellow onions cultivated worldwide [1] with a wide range of health advantages [2]. This may have a positive impact on public health given the extended consumption of these bulbs [3,4]. Like all fresh produce, onions are typically consumed raw or with very little processing, and microbial contamination of the outer layer as well as internal layers of the bulb can happen at several stages of production in the field, during curing or during postharvest operations such as packing and fresh-cutting [5,6,7]. 

Different colored onions are well known for having high flavanol contents, especially quercetin and kaempferol [8]. A comparison made on the phenolic content of four different onion scale colors found red onions containing the highest concentrations of kaempferol and quercetin [9] with skins/peels having greater numbers than their edible region because of their antimicrobial effects against soil microbes [10]. External factors like processing depend on how sensitive the phytochemical is to degradation and/or modification, the amount of time it is exposed to a processing procedure [11], and the time needed for storage [12]. Treatment types, home processing approaches, and atmospheric conditions affect the flavonoid content of onions [13,14]. Various of these phenolic compounds possess antimicrobial activity [4,6], and this antimicrobial effect is perceived as a natural defense against the survival of pathogens such as *Salmonella* in case of onion contamination [6]. In fact, foodborne illness outbreaks and recalls are less commonly linked to bulb onions than other produce commodities [15]. The antimicrobial activities of phenolic compounds, particularly against Gram-negative bacteria, have been proposed due to their ability to diffuse passively through the outer membrane mediated by electrostatic and ionic interactions and make their way into the cytoplasm, causing acidification and cell death [16,17,18]. Bulb onions are typically processed in dry environments with no water used or added on purpose. Despite all these, *Salmonella* and other pathogens can still cause contamination even when dry handling is practiced. *Salmonella* can adapt and survive for a long period due to its better heat resistance in desiccated environments [19]. In 2020, bulb onions were involved in a *Salmonella* Newport outbreak in the United States. This was the third largest *Salmonella* outbreak to have been experienced in the United States in the last three decades with 1127 total illnesses, 167 hospitalizations, and 0 deaths. A traceback investigation into this 2020 incident revealed that protocols at the dry packinghouse may have accelerated the *Salmonella* spread even though precise sources of contamination were not identified [20]. In 2021, onions in 39 states of the United States imported from Chihuahua, Mexico led to 1040 illnesses and 260 hospitalizations leading to its recall with *Salmonella* Oranienburg as the culprit [18]. These incidents were thoroughly studied [21] and showed that human pathogens can survive in onions despite the presence of compounds with strong antimicrobial activity, which may depend on other factors. However, sufficient data on the effect of environmental, genetic, and structural factors on the expression of the reported antimicrobial effect of onions could not be found. The effect of factors such as lighting and onion variety on the onion’s antimicrobial activity has been reported before [22,23]. However, other factors such as physical tissue disruption or moisture content have not yet been studied. This study focused on factors such as postharvest exposure to different types of light, the germplasm, onion variety, mode of inoculation, and moisture of the onion tissue where inoculation occurred, affecting the antimicrobial activity of bulb onions and their extracts. Based on published research, the antimicrobial effect was attributed largely to the polyphenols present in the onions [4,6]. To achieve this, challenge studies were performed to determine the effect of these factors on the fate of *Salmonella* in onion extracts as well as bulb onions from red, yellow, and white varieties. The potential role of water activity (a_w_) on the antimicrobial effect attributed to polyphenols was also tested.

## 2. Materials and Methods

### 2.1. Antimicrobial Effect of Onion Extracts on *Salmonella* Survival and Growth

#### 2.1.1. *Salmonella* Strains and Inoculum Preparation

Five strains of *Salmonella enterica* subsp. *enterica* were obtained from the Food Microbiology Laboratory’s (Texas A&M University) bacterial culture collection. These strains corresponded to serotypes: (1) Saint Paul (obtained from the FDA, isolated from a jalapeno outbreak in 2008), (2) Poona (obtained from outbreaks related to cantaloupe), (3) Michigan (obtained from tomato-related outbreak; both 2 and 3 from Dr. L. Beuchat, University of Georgia), (4) Typhimurium ATCC 700720 (originally isolated from natural sources), and (5) Enteritidis PT 30 ATCC 1045 (both 4 and 5 bought from American Type Culture Collection). Using 5 strains, each with different produce and environmental sources allowed the preparation of a cocktail with a reasonable number of strains to represent produce-related *Salmonella*. Each of these strains was independently stored in a −80 °C freezer as cryopellets (Key Products, Texas). Prior to use, one cryopellet of each strain was aseptically transferred into 10 mL tryptic soy broth (TSB, Difco, Becton Dickinson. Sparks, MD, USA) in a biosafety cabinet and incubated for 24 h at 35 °C. Following incubation, working cultures of each strain were prepared by transferring 10 µL of each isolate onto tryptic soy agar (TSA) plates (Difco), incubating 24 h at 35 °C, picking a single colony for streaking onto TSA slants, and incubating as described above. To prepare the *Salmonella* cocktail, a portion of each working culture was transferred into 10 mL TSB contained in a sterile 50 mL screw-cap conical tube and incubated 24 h at 35 °C. After incubation, the cells were washed 3 times by centrifuging the tubes at 3000× *g* at 4 °C for 15 min, then discarding the supernatant and resuspending the pellet with 10 mL PBS. After the last cell wash, all suspensions were mixed in a sterile tube and shaken in a vortex mixer for 1 min. The resulting cocktail was diluted and to achieve a target final concentration of 8.0–9.0 log CFU/mL. The final bacterial concentration was verified by colony counting on TSA plates. This suspension was used to inoculate the wells in the microplates. 

#### 2.1.2. Onion Extract Preparation

Onion cultivars Sofire (red), Carta Blanca (white), and Rio Dulciana (yellow) were exposed postharvest to red, blue, and white LED lights (Texas A&M AgriLife Research and Extension Center, Uvalde, TX, USA) and to natural light (control) under the direction of Dr. Vijay Joshi. Red and blue lights were selected due to the reported postharvest exposure to these wavelength ranges, whereas white light was selected to test the effect of exposure to a combination of all wavelengths. Additionally, Texas A&M onion germplasm 218Y, 541X, 1196, Yellow H6, 545X, 25X, 542, 1197, 1102, 231Y, 544X, and 1104 were grown in the field at the Center under the direction of Dr. Subas Malla. Both sets of onions were used to prepare extracts from the outer and inner scales to determine the effect of the potential active compounds. The sonication-assisted method was utilized as a mode of extraction based on Jang et al. [12] with slight modifications. One gram of onion skin powder of each cultivar was mixed independently with 25 mL of methanol with an extraction ratio of 1:25 *w*/*v* into beakers. All beakers were left all night at a temperature of 4 °C. Sonication (Q Sonica, Newtown, US) of samples was carried out for 10 min at 20 kHz frequency and 50% amplitude and vortexed for 5 min repetitively two times. Centrifugation was carried out for 10 min at 5000× *g* at 5 °C using a refrigerated centrifuge (3-18KS, Sigma Laborzentrifugen GmbH, Osterode am Harz, Germany). The supernatants were evaporated, and the tubes stored in a freezer (−80 °C) were used for pellet collection. Extracts were prepared by personnel in Dr. Joshi’s laboratory and were transported to the Food Microbiology Laboratory, Texas A&M University, College Station for *Salmonella* inhibition assays. Upon receiving, the extracts were stored under refrigeration. The extracts were filter-sterilized prior to use for growth inhibition testing. 

#### 2.1.3. Microtiter Broth Dilution Method

The microtiter dilution method as described by Brandt et al. [24] was followed except that no dilutions were made. Briefly, 100 μL of each of the extracts to be evaluated were pipetted into a well of a 96-well sterile microplate (Fisher Scientific, Waltham, MA, USA) and then added to 100 μL of the 5-strain *Salmonella* cocktail. This resulted in a ½ dilution (100 µL onion extract: 100 µL of *Salmonella* cocktail), which was the maximum concentration possible by this technique. In another well, 100 µL of the extract was mixed with 100 µL of PBS to serve as a baseline for the optical density (OD) measurement at 630 nm. In separate wells, sterility controls were created by mixing 100 μL of Mueller–Hinton (MH) broth with 100 μL of PBS, and growth controls were created by mixing 100 μL of the *Salmonella* cocktail with PBS. A 24 h process of recording using a spectrophotometer (Cytation 5, CA) was set for hourly OD measurements at 37 °C at a wavelength of 630 nm (OD630) to monitor the growth of *Salmonella* in the wells mentioned above. The positive change (increase) in OD over 24 h incubation (∆-OD) was used to determine growth inhibition. ∆-OD values ≤ 0.05 were regarded as complete growth inhibition [25].

### 2.2. Fate of *Salmonella* Serotype Newport on Red, White, and Yellow Onion Varieties

#### 2.2.1. *Salmonella* Newport Strains and Inoculum Preparation

For the tests on onion bulbs, *Salmonella enterica* subsp. *enterica* serotype Newport strains CC1070, CC1071, CC1072, and CC1073, isolated from clinical cases within the onion-related outbreak of 2020 were provided by D. Mann (University of Georgia, U.S.A—Center of Food Safety). Upon reception, each individual strain was streaked onto xylose lysine deoxycholate (XLD) agar to check purity, and frozen stocks with glycerol were prepared and stored in a −80 °C freezer for future use. The preparation of the *Salmonella* Newport suspensions followed the same steps as described in Section 2.1.1 above. Prior to combining all strains, each suspension was diluted to achieve a target concentration of 5–6 log CFU/mL, and then mixed in a sterile tube and mixed by vortex for inoculating onions as described below.

#### 2.2.2. Procurement and Inoculation of Onion Bulbs

For the inoculated challenge studies, pre-packed red, white, and yellow onions were obtained from a local vendor a day before the experiment. They were transported to the laboratory as bagged from the store and put in plastic boxes with as few bulbs as possible in each box to prevent overcrowding and injury. Once in the lab, they were stored in alcohol-sanitized plastic containers at room temperature (25 °C) before the experiment to simulate the storage, handling, distribution, and curing process of onions. These trials were conducted in two different experiments, each with slightly different inoculation methods.

For the first experiment, 90 bulbs of each onion variety were individually marked with food-grade ink by drawing 6 squared areas of 4 × 4 cm (16 cm^2^) over the bulb surface on the outermost papery layer. This will be referred to as the outer layer. These marked surfaces were each spot-inoculated by dispensing 100 µL of the inoculum over the 16 cm^2^ area using a sterile micropipette. Another 6 squares of 16 cm^2^ were also marked and used as guides for internal inoculation, achieved by injecting 100 µL inoculum into the bulb by puncturing at the center of the square with a loaded sterile tuberculin syringe with a 0.4 × 13 mm precision needle (Becton Dickinson), inserting the entire needle length and releasing the inoculum. The depth of the puncture was determined previously to achieve inoculation at the 3rd scale inside the bulb. Negative controls consisted of 100 µL of sterile PBS added superficially or by injection as described above. The inoculated bulbs and negative controls were placed in storage boxes at room temperature. Immediately after inoculation (zero time) and after 3, 6, 12, and 18 days of storage, 3 bulbs from each onion variety were collected for sampling and testing. A set of non-inoculated onions was tested to verify the absence of indigenous *Salmonella* at levels that may have interfered with the enumeration of the inoculated bacteria. In a separate trial, the effect of the inoculum level on the magnitude of reductions was tested. A similar experimental design was followed, this time using only red onions and a target concentration in the inoculum of 8–9 log/mL. 

For the second experiment, the target concentration in the inoculum was increased to enable detection, given the expected fast reduction observed in the previous experiment. The concentration in the cocktail was adjusted to a target level of 7–8 log CFU/mL. Sixty-four onion bulbs of each variety were inoculated on the outer papery layer as described above, and another 64-onion set was used to inoculate the internal scales, peeling by hand up to the third layer and individually marking with food-grade ink by drawing 3 (three) 4 × 4 cm (16 cm^2^) areas over the bulb surface. These marked surfaces were inoculated by gently placing 100 µL of the inoculum using a micropipette to prevent the inoculated suspension from running over the marked areas. The inoculated onion bulbs were then stored as described above. Directly after inoculation (zero time), and after 1, 4, and 6 days of storage, 6 bulbs from each onion variety were collected for sampling and testing. 

#### 2.2.3. Sample Collection and *Salmonella* Enumeration

During the trials using puncture inoculation, 3 onion bulbs of each onion variety were collected at each sampling time. From each bulb, six 4 × 4 cm (16 cm^2^) samples were collected from the outer scales, totaling 288 cm^2^. Sampling was achieved by excising the corresponding 16 cm^2^ area with a sterile scalpel and forceps. All samples were composited in a sterile stomacher bag with 20 mL of buffered peptone water (BPW). This volume was later used as a factor for calculating the final bacterial counts. To sample the inner scale, the external parts of the 3 onion bulbs were peeled to expose the 3rd scale under the areas that had been previously marked, which then was harvested by cutting approximately 16 cm^2^ using a sterile scalpel and forceps. The 6 inner scale samples for each bulb (288 cm^2^) were composited as described for the outer scales. For the trials involving exposed 3rd scale inoculation, 3 outer scales and the 3 exposed inner scales were excised using a sterile scalpel and forceps and were each composited in a bag containing 20 mL of BPW. Three onions of each variety were sampled separately at each sampling time. All samples were homogenized in a stomacher for 2 min, then decimal dilutions were prepared in buffered peptone water (BPW, Difco), selecting the lowest dilution to use according to the inoculum level used (dilution 10^0^ for low inoculum level and 10^1^ for higher inoculum level). All dilutions were then plated onto TSA plates, incubated at 37 °C for 2 h, and then overlaid with XLD agar to continue incubation over 18–24 h. The overlay was applied to allow resuscitation of bacteria potentially subjected to sub-lethal injury after exposure to onion tissue, which may affect the colony development on *Salmonella*-selective media such as XLD. Colonies typical of *Salmonella* were enumerated. To confirm identity as *Salmonella*, random colonies were picked, streaked onto XLD, and subjected to biochemical and serological tests following the guide by the Microbiology Laboratory Guidebook (MLG) 4.14 [26]. Since no *Salmonella* was found on control (non-inoculated) onions, it was assumed that the counted salmonellae were the same as those inoculated. The colony counts for each sample were divided by the total area composing each sample to use conventional reporting units or CFU/cm^2^. The limit of detection (LOD) of the plating method varies according to the dilutions prepared for plating, which are selected based on the expected bacterial concentration in the food. In our case, the LOD for the first experiment was −1.2 log CFU/cm^2^, whereas for the second experiment, the LOD was 1.1 log CFU/cm^2^.

### 2.3. Measurement of Water Activity

Onion layers from bulbs corresponding to the 3 varieties tested (red, white, and yellow) were collected from layers at various. For each variety, 3 square samples from the outer papery layer (skin) and the 1st, 2nd, 3rd, and 4th internal layers (a total of 15 samples per onion variety) were excised from 3 bulbs in a total of 3 independent trials. The samples were collected by delimiting a 4 × 4 cm square and excising using a sterile scalpel and forceps. Each sample was chopped into small pieces by use of a scalpel and subjected to water activity (a_w_) measurement using an AquaLab PRE water activity analyzer (Decagon, Pullman, WA, USA). The a_w_ measurements were tabulated and subjected to statistical analysis as described below.

A 4 × 4 cm (16 cm^2^) area was cut out of bulbs of the onion cultivars (red, white, and yellow) from the outer papery layer (skin), and the 1st, 2nd, 3rd, and 4th layers and chopped into small pieces by use of a scalpel after that to be used for water activity (a_w_) measurement using the AquaLab PRE water activity analyzer (METER Group, WA, USA).

### 2.4. Statistical Analysis

All experiments described above were repeated in 3 independent trials, each using 3 replicate samples per treatment. For the onion extracts trials, optical density (OD) data were obtained by the difference between the actual OD and the baseline OD, and the results were analyzed by use of one-way ANOVA using the Least Square Means method in JMP (JMP Pro, v16.0, SAS Institute Inc., Cary, NC, USA). For the survival studies, microbial counts were converted into log values. To facilitate statistical analysis counts, samples with no detectable colonies in any of the dilutions were assigned a CFU/cm^2^ value equal to one-half of the detection limit, and then, this number was converted to log value as with the rest of the values. The count was calculated in CFU/cm^2^ to be consistent with the total area inoculated per onion bulb. Values of OD, log CFU/cm^2^, and a_w_ were separately analyzed by multifactorial analysis of variance in the Fit Model function of JMP, with LS Means Tukey HSD at *p* < 0.05 for mean separation to compare the effects of each onion cultivar on the survival of the *Salmonella* cocktail over the 6 and 18-day storage period and on the moisture of the onion scales at varying depths.

## 3. Results

### 3.1. Antimicrobial Effect of Onion Extracts on Salmonella Survival and Growth

#### 3.1.1. Effect of Various Exposed Lights During Onion Growth on the Inhibition of Salmonella by Extracts of Red, White, and Yellow Onion Varieties

**Figure 1:**
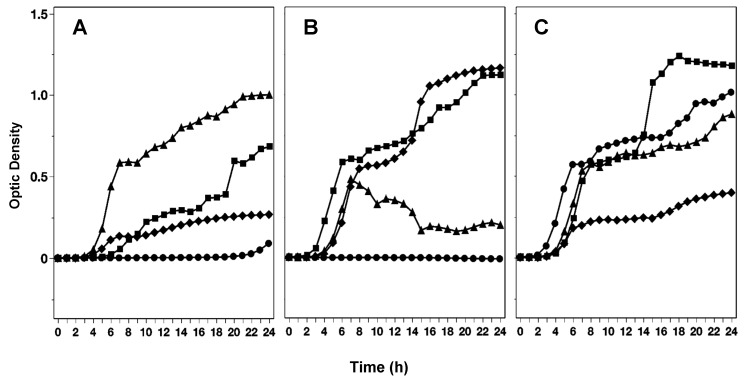
Growth and inhibition of *Salmonella* in extracts of red (**A**), white (**B**), and yellow (**C**) onions made from bulbs exposed to natural light (diamonds) or artificial blue (circles), red (triangles), white (squares) light during curing.

The ability of *Salmonella* to grow in the presence of red, white, or yellow onion extracts was affected by the type of light to which the onions were exposed during curing. Figure 1 shows how the growth or survival of a 5-strain *Salmonella* cocktail differed within extracts of the same onion cultivar when blue, red, white, or natural light was used to illuminate the onions postharvest (at curing). Under blue light, extracts of red and white onion cultivars significantly (*p* < 0.05) hindered the growth of *Salmonella* as compared to yellow. Although in most of the extracts, the growth of *Salmonella* was not completely inhibited, there were significant differences in the growth rates as affected by the extract.

For the red light, onion extracts of the white variety significantly (*p* < 0.05) slowed down the growth of *Salmonella* compared to red and white. White light was associated with significantly slower growth of *Salmonella* in red onion extracts than yellow and white onion extracts. In contrast, when onions were cured under natural light, the red onion extracts produced the strongest effect against *Salmonella*, followed by yellow onion extracts, while the growth was not inhibited or retarded by the extracts of white onions. 

#### 3.1.2. Impact of Various Onion Genotypes and Their Layers on Salmonella Growth

Data in Table 1 show the ∆-OD (change in OD over 24 h incubation) for *Salmonella* in extracts of onions corresponding to 12 genotypes. Except for Genotype 1104, the extracts tested could not prevent *Salmonella* growth regardless of whether they were made from the outer or inner onion scales. In Genotype 1104, belonging to red onion germplasm, the extract made from the outer scales showed a mean OD of −0.0025, much lower than the ≤0.05. These data provide little information due to the great variability within samples. As such, we cannot conclude that genotype 1104 in fact possessed greater antimicrobial activity than the other genotypes, or that the extracts made from outer layers were more antimicrobial than those prepared from internal scales.

### 3.2. The Fate of Salmonella Serotype Newport on Yellow, White, and Red Onion Bulbs During Storage at Room Temperature

*Salmonella* Survival on the Outer and the Inner Onion Layers. Generally, all onion cultivars’ outer papery skin layers exerted a significantly greater inhibitory effect against *Salmonella* Newport in the 18 days of the experiment. No colonies were detected on the lowest dilution of uninoculated onion samples; the detection limit was −0.5 log CFU/cm^2^, showing no evidence of inherent *Salmonella* in onions.

**Figure 2:**
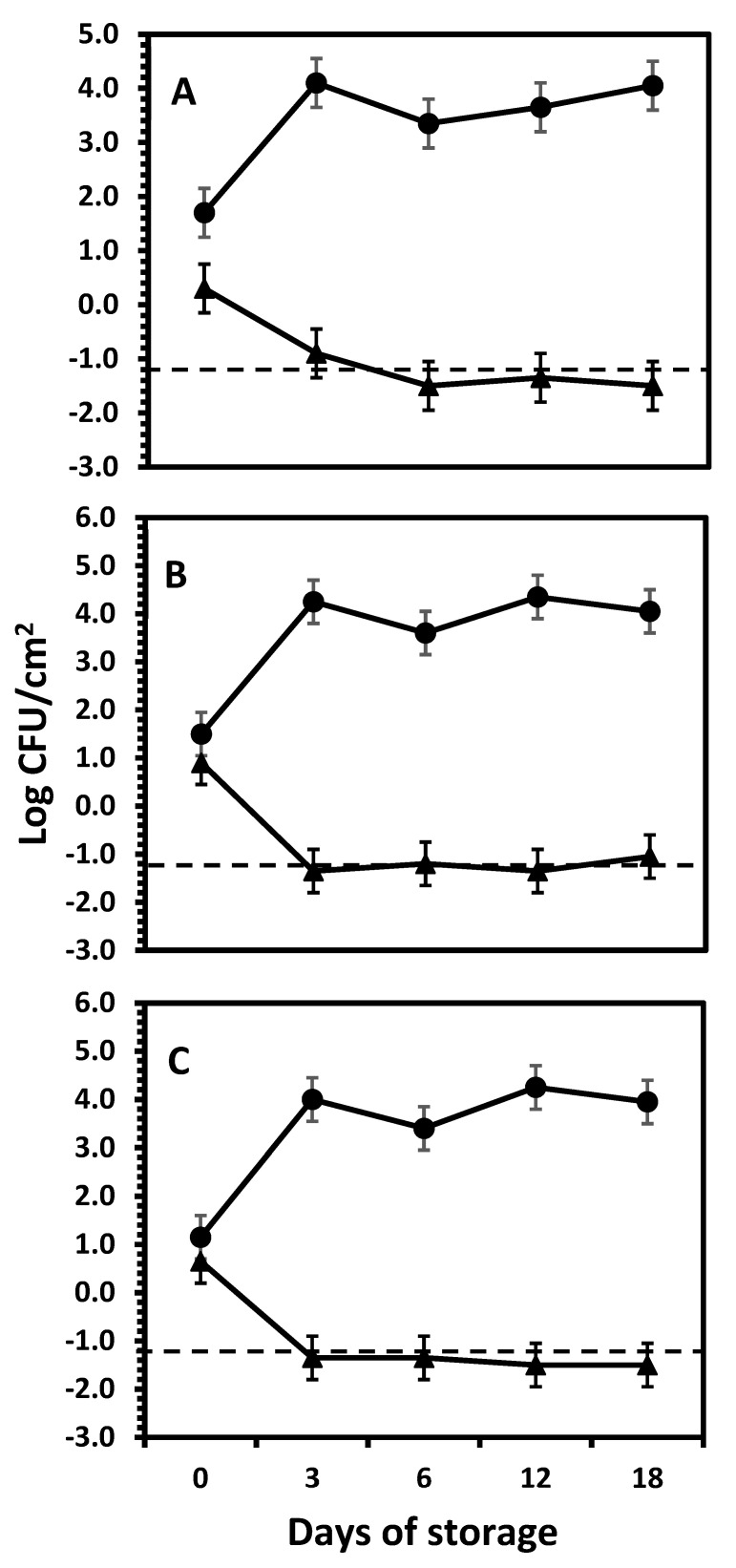
Growth of *Salmonella* Newport cocktail on the outer papery skin layers (triangles) and in the inner layers (circles) of red (**A**), white (**B**), and yellow (**C**) bulb onions stored in containers and held at room temperature (25 °C). The lines crossing the chart show the limit of detection of the counting method (−1.2 log CFU/cm^2^). Each data point represents the mean of at least 9 tested samples and the error bars represent standard error (SE). There were no differences in counts between onion varieties (*p* ≥ 0.05).

For outer layers (triangled markers in Figure 2), in all instances, the *Salmonella* population was significantly reduced (*p* < 0.05) within the first 3 days of storage in all onion cultivars. The initial *Salmonella* mean populations of the outer scales of red, white, and yellow onions on day 0 between 0.3–1.5 log CFU/cm^2^, and experienced a detrimental growth decline within the first 3 days of storage, to −0.9 log CFU/cm^2^ for red onion, whereas in white and yellow onions, these counts were at or below the detection limit (<−1.2 log CFU/cm^2^). The mean decline in the *Salmonella* population from 0 to 3 days was 1.2, >2.1, and >1.9 log CFU/cm^2^ in red, white, and yellow onions, respectively, since all counts were reduced to levels below the limit of detection of the plate count method. No significant differences (*p* ≥ 0.05) were found between the 3 onion varieties tested. These results were confirmed during trials repeating the same procedure on red onions but increasing the inoculum level to ~5 log CFU/cm^2^. Again, the counts of *Salmonella* Newport inoculated on the outer papery layer were reduced to undetectable levels within the first 3 days of storage, remaining undetectable for the remaining 18 days of storage. The magnitude of reduction was >3.7 log cycles, confirming the strong and rapid antimicrobial effect of the external layer of the onion bulb. In contrast, the internal layers inoculated by puncturing supported the growth of this organism, to a maximum increase of 1.7 log cycles at day 12 of storage.

From these results, it can be concluded that the decline rate of *Salmonella* Newport on the outer papery layers of the 3 onion varieties was similar for all onion varieties, going from the initial numbers of ~1 log CFU/cm^2^ to not detectable within 3 days of storage. There was no further significant change (*p* ≥ 0.05) in the *Salmonella* mean population in all onion varieties from day 6–18, which remained below detection levels during the rest of the storage period. The low numbers reported here are the result of the conversion to CFU/cm^2^. However, during data analysis, the variances and standard errors remained unchanged with or without data conversion, as expected. The decision to present results in CFU/cm^2^ intended to show data in a conventional and understandable type of units, without affecting the accuracy of the conclusions drawn from the data analysis.

In contrast, for the syringing/wounding/bruising inoculation method (circles in Figure 2), *Salmonella* Newport underwent a significantly (*p* < 0.05) different pattern as compared to the outer layers; *Salmonella* Newport was able to grow in the inner layers of the three onion cultivars (Figure 2). In the first 3 days, *S*. Newport experienced an increase in population by 2.3, 2.6, and 2.6 log cycles in the inner layers of red, white, and yellow onions, respectively. Typically, in the 18-day storage time, the growth of *Salmonella* Newport in the inner layers of red onions followed a constant but small trend. The initial mean population of *Salmonella* Newport after inoculation (0 days of storage) in red, white, and yellow was 1.7, 1.5, and 1.2 log CFU/cm^2^, respectively.

After 3 days, the counts of *Salmonella* Newport underwent a significant (*p* < 0.05) increase to 4.1, 4.3, and 4.0 log CFU/cm^2^ for red, white, and yellow onions, respectively. At this time, *Salmonella* Newport seemed to reach the stationary phase, with a calculated maximum population increase of 2.4, 2.6, and 2.8 log cycles over 18 days of storage for red, white, and yellow onions, respectively. No differences (*p* ≥ 0.05) in the growth patterns were observed between all onion varieties when inoculation was achieved by puncturing.

**Figure 3:**
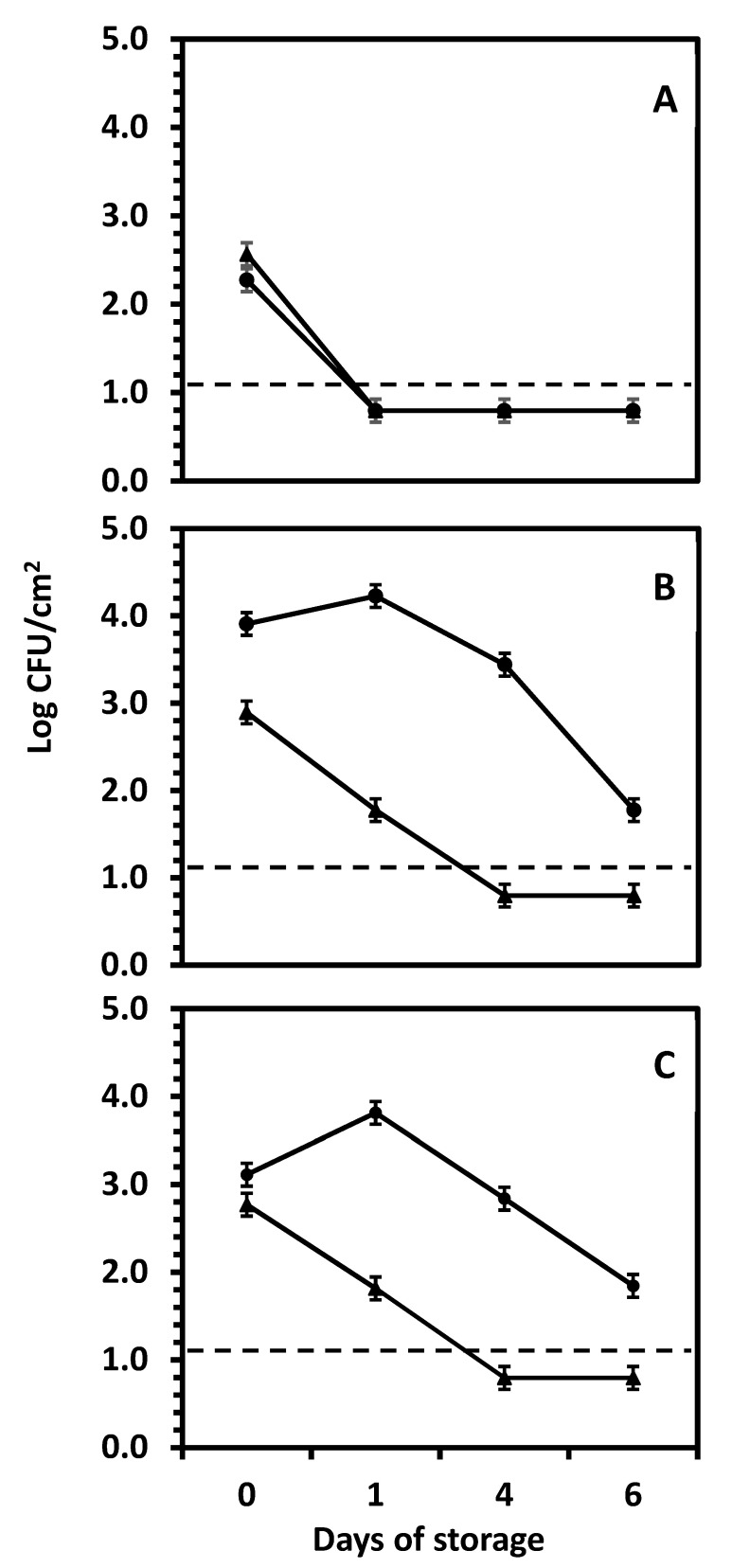
Reduction of *Salmonella* Newport on outer (triangles) and inner (circles) layers of red (**A**), white (**B**), and yellow (**C**) onions during storage at room temperature. The dotted lines crossing the chart show the limit of detection of the counting method (1.1 log CFU/cm^2^) and the error bars represent standard error (SE). Each data point represents the mean of at least 9 tested samples.

In a subsequent experiment, the survival of *Salmonella* Newport on the outer vs. inner layers of red, white, and yellow onions was tested. The data in Figure 3 show significant (*p* < 0.05) effects of the location of the onion layer, time of storage, and onion variety on the survival of *Salmonella* Newport over a 6-day storage period. Unlike wounding (see Figure 2), where *Salmonella* Newport grew rapidly, the exposure of the 3rd scale followed by surface inoculation resulted in a reduction with various decline rates during storage, depending on the onion variety. In contrast, the outer (papery) layers did not permit the growth of this pathogen, with varying rates of decline depending on the onion variety. Red onions (Figure 3A) showed a sharp decline to below the limit of detection within one day regardless of the layer (outer or inner, *p* ≥ 0.05), where *Salmonella* Newport was significantly (*p* < 0.05) reduced by >2.5–>2.8 log cycles by day 1 (reduction to nondetectable levels), remaining unchanged until the end of the storage time. Contrarily, the outer layers of yellow and white onions experienced nearly the same *Salmonella* Newport survival pattern, showing a gradual decrease to below the detection limit within 4 days. The reason why there are numbers reported below the detection limit is that some samples yielded detectable counts, but the mean counts of all repetitions and replicates were still below the limit of detection. White and yellow onions’ death rates were much slower than red onions (*p* < 0.05). In contrast to the previous experiment (see Figure 2), where *Salmonella* Newport grew inside the onions within 3 days when inoculated by wounding with a needle, exposing the inner tissue by peeling and then surface inoculating the 3rd inner scale resulted in small but significant (*p* < 0.05) growth of *Salmonella* Newport during the first day of storage, to continue with a slow decline over 6 days of storage on white and yellow onions (Figure 3B,C). On the inner scales, there were no differences (*p* ≥ 0.05) in the *Salmonella* Newport populations between white vs. yellow onions during the first 4 days of storage. At 6 days, the populations were significantly lower (*p* < 0.05) than at 0, 1, or 4 days, with a final reduction of 2.1 and 1.3 log cycles on the inner scales of white and yellow onions, respectively. The behavior on the outer papery layers of the 3 onion varieties was consistent in the experiments shown in Figure 2 and Figure 3, which show that the outer papery layers of onions express greater antimicrobial activity than the internal tissue of the onion bulb. However, when such internal tissue was disrupted (wounding by the inoculating needle, Figure 2) *Salmonella* Newport grew actively, while the non-wounded surface of the internal scales allowed only minimal growth followed by a slow reduction.

Table 2 shows the a_w_ of various layers of red, white, and yellow onions removed from fresh onion bulbs. The a_w_ of the outer layers significantly differed (*p* < 0.05) from that of the inner layers. Generally, the red variety showed lower a_w,_ particularly in comparison to white, while yellow onions showed variations in similarities with either red or white onions. However, the most critical finding was the gradual increase in a_w_ as the layers were more profound in the onion bulb. In all onion varieties, the deeper the layer location, the higher the a_w_. On the outer layer, a_w_ values were <0.6, which is often regarded as the a_w_ threshold for supporting microbial growth. This explains why the outer layers consistently inhibited the growth of *Salmonella*. However, the expected higher concentration of phenolics in the outer layers may not be sufficient to explain the reduction of *Salmonella* Newport (see Figure 2 and Figure 3) since the absence of moisture may also interfere with the transfer of molecules to the bacterial cells. The 1st layer after the outer papery skin also showed significant differences (*p* < 0.05) across the three varieties with mean values of 0.764 ± 0.023, 0.904 ± 0.042, and 0.863 ± 0.027 for red, white, and yellow, respectively. The mean water activity of the red variety (0.836 ± 0.023) in the 2nd layer after the outer papery layer was significantly different (*p* < 0.05) than the white (0.909 ± 0.001) and yellow (0.895 ± 0.017) varieties. Similarly, in the 3rd layer, the red onion was significantly different (*p* < 0.05) from the yellow and white types with a_w_ mean of 0.897 ± 0.02 (red), 0.928 ± 0.003 (white), and 0.921 ± 0.015 (yellow). There was a significant difference (*p* < 0.05) in the 4th layer after the outer papery layer in all onion varieties with red recording the lowest mean a_w_ of 0.961 ± 0.011, followed by yellow with 0.972 ± 0.001 and white with 0.997 ± 0.003. Across the rows in Table 2, the a_w_ for each variety increased with the depth of the layer. All varieties consistently increased in a_w_ from 0.51–0.58 on the outer layer to 0.96–0.99. The white onions consistently showed a_w_ > 0.90 since the 1st layer after the outer, whereas yellow onions showed a_w_ values > 0.90 since the 3rd layer after the outer. 

## 4. Discussion

The observation that the onion variety had an effect on the antimicrobial activity of onion extracts against *Salmonella* supports a bulk of previous research on phenolic compounds was not surprising. Given the recognized antimicrobial activity of phenolic compounds, which are abundant in onions [4,6,16,17,18], it is reasonable to assume that any inhibition of growth or reduction in *Salmonella* observed in this study be attributed to the polyphenols, mostly quercetin, the richest polyphenol in onions [27,28]. Quercetin compounds (the primary flavonoids in onions) are linked to plant disease resistance [27], and the quercetin content of red onions has been shown to differ significantly from the other types, while yellow, white, and sweet onions were not significantly different [28]. In addition to their antimicrobial properties, onion extracts and quercetin also interfere with quorum sensing, which controls violacein synthesis and swarming motility [29]. According to earlier studies, onion oil and bulb extracts had a major impact on various microorganisms [29,30], and the structure of quercetin’s flavone skeleton may aid in its inhibitory action [31].

The antimicrobial activity was also affected by the exposure of the onions to various types of light during curing. Many modern produce storage technologies use light manipulation (including LED lights) to prolong shelf life and preserve nutritional quality. The effect of light stress on seed germination and plant growth has been demonstrated in various plants including onions [32,33]. This effect may be different depending on the light wavelength used for plant exposure [29]. Different wavelengths of light affect the physiological processes, including the production of antimicrobial compounds like phenolic acids and flavonoids. This is achieved through various mechanisms including the phenylpropanoid metabolic pathway, which produces metabolites that scavenge oxidating radicals and regulates energy dissipation in plants. In particular, chlorogenic acid acts as a low molecular weight antioxidant in plants, providing protection during light stress [34]. Mechanisms by which visible or UV light could manipulate phenolics across plant species have been extensively reviewed [22,29,30]. Particularly, light of short wavelength (blue, UV) can increase antioxidants such as phenols, flavonoids, and flavones in various plants, including onions [34,35], and several studies have repeatedly pointed out quercetin’s impact on *Salmonella* and other enteric pathogens [31,35]. Blue light (wavelengths around 450–495 nm) influences the production of antioxidants like flavonoids and anthocyanins, which are associated with potential health benefits, including anti-inflammatory and antioxidant properties. Blue light has been proven to significantly reduce the viability of various bacterial and fungal cells, including *Salmonella*, on various surfaces, including fresh produce, through the generation of reactive oxygen species such as singlet oxygen and free radicals, which could disrupt the cellular structures of pathogens like *Salmonella*, leading to oxidative stress and eventual cell death [36,37,38,39]. Red light (wavelengths around 620–750 nm) can trigger the production of certain phytoalexins and secondary metabolites and the accumulation of carotenoids like beta-carotene, which have antioxidant properties [40,41]. White light, a mixture of blue, red, and green light wavelengths, can positively and negatively affect microbial growth by stimulating the production of antimicrobial compounds. It has been suggested that high levels of carotenoids in yellow onions could absorb most of the blue light, limiting their ability to stimulate quercetin biosynthesis, which has potent anti-salmonella activities [29,42]. In contrast, anthocyanins dominating red onions are likely more responsive to blue light to trigger quercetin metabolism [43,44]. The observation that white onions were not inhibited when onions were exposed to natural light may be explained by the fact that white onions have a lower flavonoid content in comparison to colored onions [27]. Although limited studies have been performed on onions per se, studies on other crops have shown that quercetin synthesis under blue light is generally more pronounced in red than in yellow fruits because of pigmentation. Regarding the effect of the genotype on the antimicrobial effect of onion extracts, the variability of data and the low antimicrobial effect shown overall did not permit any conclusion to be drawn. Further research is warranted to determine whether the genotype of onion determines the antimicrobial effect, most likely resulting from higher concentrations of polyphenols.

The challenge studies testing the survival or growth of *Salmonella* Newport inoculated on the surface and inside onion bulbs, attempted to simulate the dynamics of this pathogen when transferred to the onion surface by direct contact with contaminated surfaces, internalizing in the onion bulbs during produce handling, or plant damage by insects and plant pathogens, producing wounds that may serve as entry points for human pathogens [45,46,47] or pathogenic bacterial diseases that tend to occur close to harvest [48], facilitating the entry of human pathogens. When pathogens are internalized, the concentration of polyphenols in response to such damages is not always associated with activating defensive machinery but often compromises the normal functioning of stomata, distribution of photoassimilates, modification of cell walls, water, and ion imbalance [49], all conducive to the survival of human pathogens. Results show that the outer layer had a significant inhibitory impact on the survival of *Salmonella* Newport in the 3 onion varieties. This is consistent with other studies on the inoculation of onion skin [50,51,52] and this effect is attributed to the phenolic contents being high on the skin of the onion [52,53,54]. In fact, onion waste is recognized as an important source of naturally occurring bioactive chemicals [10,55,56]. In this study, the inner layers of yellow, white, and red onion varieties supported the growth of *Salmonella* Newport in white and yellow varieties, whereas red onions showed consistent suppression of *Salmonella* Newport growth during storage. The literature discussed here makes it clear that red onions have a higher concentration of polyphenols [23,27,57] and that the onion’s outer papery skin contains higher concentrations of polyphenols [23,55,56,58]. In fact, Kim and Kim [59] reported that the quercetin concentration in onions gradually decreases between the inedible papery skin and the bulb’s core.

The findings of this study support earlier reports that red onions possess greater antimicrobial capacity than other onion varieties, which is attributed to the reported higher concentrations of specific polyphenols [23]. This may explain why *Salmonella* Newport could not grow or survive in red onions as much as they were able to in white and yellow onion tissues. The antimicrobial activity of red onions reported by Sharma et al. [23] is generally consistent with our results. 

Regarding the greater antimicrobial effect shown by the outer papery layer of onions compared to the inner layers, not many studies were available on the influence of onion skins on the growth of *Salmonella*. However, it has been elucidated that onion peel contains roughly 3–5 times more isolated phenolic compounds and quercetin than the edible portion of the onion [60], supporting the assumption that the antimicrobial activity observed in this study may be attributed to the effect of these compounds. Furthermore, extracts prepared from the edible components of onions have exhibited decreased antimicrobial efficacy against these bacteria, including no growth inhibition for *E. coli* [23,49]. With regards to the reduction of *Salmonella* Newport, when inoculated on the outer papery layer of onions, some reports support the findings in this study. Antimicrobial activity against *S. aureus* and *E. coli* was observed in red onion bulbs’ outer layers (skin) extracted by direct maceration [53], while Kumar et al. [50] found the largest quantities of flavonoids and phenolics in onion skin when compared to edible onion flesh. The higher concentration of phenolic compounds in the outer papery layer of onions may be facilitated by the drying process that starts from the curing step. By drying, the polyphenols are expected to become more concentrated in the skin, as it collapses due to water loss. In contrast, the internal tissues would have a higher water content, which may dilute the polyphenols or accelerate their degradation.

Another important observation was the rapid growth of *Salmonella* Newport when inoculated by creating a wound with a needle and syringe. Wounding or bruising could occur throughout the supply chain and physical damage is reported as one of the major mechanisms for infection with plant pathogens [60]. This may be extended to human pathogens that may contaminate onions at pre and postharvest stages. Factors promoting produce bruising and how physical damage favors pathogen internalization have also been described in other commodities [61,62,63,64]. It is likely that the same mechanisms for tissue damage described for tomatoes by Tokarskyy et al. [63] would operate in other produce commodities, such as onions. In onions, deep bruising may release large amounts of liquid, favoring microbial growth, which could explain why *Salmonella* Newport experienced a decline when onions were peeled in our study compared to when the injury was imparted by syringing. The higher a_w_ observed with an increase in scale depth across the three onion varieties could aid in explaining the growth of *Salmonella* in the inner layers upon wounding/syringing, especially in yellow and white varieties.

To the best of our knowledge, this paper is the first to study the a_w_ of onions at various depths at room temperature.

## 5. Conclusions

The highlights of this study focused on the effects of various factors on the survival or growth of *Salmonella* in onion extracts and bulbs. Although no attempts were made to study the effect of phenolic compounds from onion, the data showing the antimicrobial activity of onion extracts and tissues against *Salmonella* underlines the need for further research. This is especially urgent since under some conditions, *Salmonella* showed the ability to grow and since the antimicrobial effect of onions is attributed to the phenolic compounds, there is a need for a mechanistic description of synergism between phenolics and possibly other compounds, as well as other biochemical or physicochemical interactions that result in the expression of antimicrobial activity in onions. The inhibition not only of *Salmonella* but possibly of other biofilms associated with foodborne pathogens underlines the need for more detailed exploration of their potential to interfere with quorum sensing to control biofilm and toxin production in bacteria. Further research is needed to harness the dual role of onion phenolics as nutraceuticals and environmentally friendly antibacterial tools.

In this study, comparing the results of the trials with onion extracts with those where onion bulbs were directly inoculated, it becomes apparent that these two approaches may provide different points of view on the antimicrobial activity of onions. The challenge studies, which may represent more realistic onion consumption scenarios, revealed that outer layers consistently inhibited *Salmonella* growth while inner layers showed increased bacterial populations and that the more severe the tissue disruption, the higher the growth rate. In addition, the a_w_ increased consistently with the depth of the onion scales. Although the relation between red onion variety and rapid decline of *Salmonella* was expected due to the known higher concentration of polyphenols in the red onions, these trials show that the ability of *Salmonella* to grow in and on onions may be multifactorial and not exclusively related to the presence of polyphenols with antimicrobial activity but at least, a dual effect between water activity and polyphenols. While more research should be conducted to elucidate all factors in *Salmonella* inhibition by onions, this research supports the importance of maintaining adequate postharvest product handling practices. Preventing bruising of onion bulbs seems to be paramount in preventing the growth or survival of *Salmonella* in bulb onions. We recommend that it is fundamental for onion processors and consumers to adhere to careful and/or strict handling procedures of onions given their delicate nature to avoid damage/injuries/wounding which could exacerbate the attachment and internalization process of *Salmonella* into the internal layers of onions resulting to eventual growth.

## Figures and Tables

**Table 1:** Growth of *Salmonella* in the presence of extracts of different genotypes of onion.

	Mean ∆OD ^a^ ± STDEV by Location of Scales Used for Extracts	
Genotype	Outer	Inner
25X	0.62 ± 0.11 AB ^b^	0.97 ± 0.35 AB
218Y	0.85 ± 0.38 AB	0.67 ± 0.02 AB
231Y	0.60 ± 0.02 AB	0.70 ± 0.22 AB
541X	0.76 ± 0.20 AB	0.68 ± 0.04 AB
542	0.93 ± 0.38 AB	0.33 ± 0.47 AB
544X	0.82 ± 0.01 AB	0.80 ± 0.01 AB
545X	0.88 ± 0.43 A	1.14 ± 0.03 AB
1102	0.83 ± 0.37 AB	1.08 ± 0.02 A
1104	−0.0025 ± 0.01 B	0.26 ± 0.36 AB
1196	0.64 ± 0.03 AB	0.92 ± 0.34 AB
1197	0.87 ± 0.35 AB	0.58 ± 0.06 AB
YELLOW H6	0.67 ± 0.03 A	1.17 ± 0.03 AB

^a^ ∆OD represents the change in OD reading at *λ* 630 for each extract and is calculated by subtracting the OD at zero time from the OD observed after 24 h incubation period. A ∆OD of ≤0.05 would be considered as having no *Salmonella* growth. ^b^ Means followed by the same letter are not significantly different (*p* ≥ 0.05). Each mean represents the OD values obtained from 9 analytical values.

**Table 2:** The water activity of red, white, and yellow onion layers at various depths at 25 °C.

			Layer		
Variety	Outer Papery	1st After Outer	2nd After Outer	3rd After Outer	4th After Outer
Red	0.509 ± 0.032 A ^a^	0.764 ± 0.023 C	0.836 ± 0.023 G	0.897 ± 0.024 E	0.961 ± 0.011 F ^b^
White	0.583 ± 0.003 B	0.904 ± 0.042 E	0.909 ± 0.001 E	0.928 ± 0.003 E	0.997 ± 0.003 G
Yellow	0.525 ± 0.011 A	0.863 ± 0.027 D	0.895 ± 0.017 E	0.921 ± 0.015 E	0.972 ± 0.001 F

^a^ Water activity (a_w_) values are means ± standard error. ^b^ Within columns and rows, means with different letters are significantly different (*p* < 0.05).

## Data Availability

The original data presented in the study are openly available in the Texas Data Repository at https://doi.org/10.18738/T8/CFAWKM and https://doi.org/10.18738/T8/W8ZHFB.
